# Prevalence of pineal gland calcification: systematic review and meta-analysis

**DOI:** 10.1186/s13643-023-02205-5

**Published:** 2023-03-06

**Authors:** Daniel Gashaneh Belay, Misganaw Gebrie Worku

**Affiliations:** 1 Department of Human Anatomy, College of Medicine and Health Science, School of Medicine, University of Gondar, Gondar, Ethiopia; 2 Department of Epidemiology and Biostatistics, Institute of Public Health, College of Medicine and Health sciences, University of Gondar, Gondar, Ethiopia

**Keywords:** Pineal gland, Melatonin, Calcification, Risk factors

## Abstract

**Background:**

Pineal gland calcification is the formation of corpora arenacea predominantly composed of calcium and phosphorus. It plays an important role in regulating the light/dark circadian changes to synchronize their daily physiological activities like feeding, metabolism, reproduction, and sleep through the secretion of melatonin. Therefore, this study aimed to assess the pooled prevalence of pineal gland calcification.

**Methods:**

A systematic review was done using published research articles from different electronic databases. Cross-sectional studies were included for systematic review and only studies conducted on the human population were included for quantitative analysis. Published articles were selected by assessing the title and abstract for relevance to the review objectives. Finally, the full text was retrieved for further assessment.

**Results:**

The pooled prevalence of pineal gland calcification was 61.65% [95% CI: 52.81, 70.49], with a heterogeneity of *I*^2^ = 97.7%, *P* ≤ 0.001. According to the qualitative analysis, an increase in age, male sex, and white ethnicity are the major socio-demographic characteristics that increase the prevalence of pineal gland calcification.

**Conclusion:**

The pooled prevalence of pineal gland calcification was higher compared with reports from previous studies. Different studies reported pineal gland calcification was most prevalent in the adult population compared with the pediatric age groups. According to the qualitative analysis, an increase in age, male sex, and white ethnicity are the major socio-demographic characteristics that increase the prevalence of pineal gland calcification.

## Background

The pineal gland is a neuroendocrine organ that regulates daily body rhythm by the secretion of melatonin [1]. It is the principal seat of the soul and the place in which all thoughts are formed [2]. The pineal gland is the part of the epithalamus, located at the posterior wall of the third ventricle occupying the space between the corpus callosum and the superior colliculi [3–6]. Different studies report that the pineal gland grows in size from birth until 2 years of age and then remains constant between 2 and 20 years of age [5–7]. Unlike most parts of the brain, the pineal gland is located outside of the blood–brain barrier (BBB) [8, 9]. The pineal gland consists of different cell types in addition to pinealocytes, which include interstitial cells, perivascular phagocytes, pineal neurons, and peptidergic neuron-like cells [10, 11]. It plays an important role in regulating the light/dark circadian changes to synchronize the physiological activities [2, 4, 5, 8], cancer inhibition [4, 5], neuroprotector antioxidant [4, 8], and induction of the endocrine activity of the hypothalamus, pituitary, ovaries, and testis [3]. Melatonin act as negative feedback to the biological clock, the suprachiasmatic nucleus (SCN), that regulates the circadian rhythm of the body. The SCN sends signals to all the organs synchronizing the day-night cycle like sleep, lowering of body temperature, and blood pressure at night time, which leads them to function at the proper time [9, 12]. Pineal gland calcification (PGC) is the formation of corpora arenacea, composed of mainly calcium and phosphorus with a small contribution of magnesium and strontium [3, 5]. Pineal gland calcification presents histologically from fetal life to adulthood, which increases in number and size with aging [1, 13, 14]. There are two types of pineal gland calcification based on the affected area of the gland, intra-pineal calcification present within the parenchyma of the gland and extra-pineal calcification within the capsule of the gland common among old age groups [3, 15, 16]. Pineal gland calcification can also be pathological or physiological depending on the causes of calcification [16]. Physiological calcifications are unaccompanied by any evidence of disease, asymptomatic, and detected incidentally by neuroimaging [17, 18], whereas pathological calcifications are extremely rare and occurred due to any diseases or abnormality in the form of developmental, reactive, neoplastic, and vascular abnormalities [19]. Other pathological conditions like neurodegenerative diseases, including Alzheimer’s disease, autism, migraine, chronic primary insomnia, and stroke, can also be associated with pineal gland calcification [2, 9]. Genetic and environmental factors, which increases pineal gland calcification, are male gender, low altitude, and low intensity of sunlight exposure [5, 9, 14]. The prevalence of pineal glad calcification ranges from 58.8 to 76% across different countries [14, 16, 20, 21]. Currently, PGC is an important marker for neurologists and neuroradiologists and is an important radiological shadow suggesting the presence of a space-occupying lesion in an intracranial cavity [3]. Since pineal gland calcification had a strong association with different pathological conditions, genetic and environmental problems, it is important to have an appropriate information on its magnitude and geographic distribution. So, this study aims to assess the pooled prevalence of pineal gland calcification.

## Methods

### Design and protocol development

This systematic review and meta-analysis were conducted and reported according to the Preferred Reporting Items for Systematic Reviews and Meta-Analysis (PRISMA). A protocol was registered in the International Prospective Register of Systematic Reviews database (PROSPERO, CRD42020200424).

### Searching strategy

Published research works are accessed using computerized search engines such as PubMed, direct Google, Google Scholar, and Cochrane library. Published papers in different countries were identified and all the available articles entitled prevalence and associated factors of pineal gland calcification were included. The search language was limited to English. Published papers were searched using keywords, such as pineal gland, prevalence, pineal gland calcification, and associated factors, and only published articles were included in the meta-analysis. The title and abstract of each study were critically reviewed by two researchers and most of the retrieved articles were excluded here. The full documents were thoroughly read and reread to include the paper for quantitative analysis. Finally, only 8 studies that meet the inclusion criteria were included for the quantitative analysis.

### Criteria for inclusion and exclusion

For this study, we included population-based studies, cross-sectional based studies, and studies that provide sufficient information on sample size and prevalence of pineal gland calcification. The quality of the articles was assessed by sample size, the aim of the study, measured variables, and study design. Therefore, all articles, which used cross-sectional based studies and report the prevalence of pineal gland calcification were included. Studies conducted at a specific population group and those done at a specific age group were excluded as it is not representative of the general population. For multiple studies on the same population, only the study that reported the most detailed data was included.

### Data extraction and quality assessment

All articles searched from 12/07/2020 to 26/08/2020 using different electronic databases were combined in Endnote and duplicates were removed. Two researchers (MG and DG) independently screened the titles and abstracts and reviewed the full text of the eligible citations. For each included study, two researchers (MG and DG) independently extracted the following information: general information (e.g., first author, title, journal, and publication year), study characteristics (including study period, study area, study design, sample source, sample selection method, and sample size), and all possible participant information. Only when reviewers agreed was the study included in the meta-analysis. Textual narrative synthesis and thematic synthesis methods were used to extract and generate important qualitative findings. The quality of the study included was assessed by Newcastle–Ottawa scale (NOS) quality assessment tools for cross-sectional studies.

### Statistical analysis

We used a systematic analysis approach to calculate the pooled estimate of pineal gland calcification from all eligible studies. A random-effect model was selected to summarize the prevalence of pineal gland calcification using statistical tests for heterogeneity. Heterogeneity among studies was assessed using Cochran’s *Q* test and *I*^2^ statistic, which shows the percentage of variation across studies (with values of 25%, 50%, and 75% indicating low, moderate, and high degrees of heterogeneity, respectively) [22, 23]. Sensitivity analysis (i.e., recalculating the pooled estimate by omitting studies with low scores) was performed to assess the influence of any study on the pooled estimate. Publication bias was assessed using Egger’s test. The significance level was set at a *P*-value of less than 0.05. All statistical analyses were performed using Stata version 11.0 and Excel 2013.

## Results

### Description of studies

**Fig. 1:**
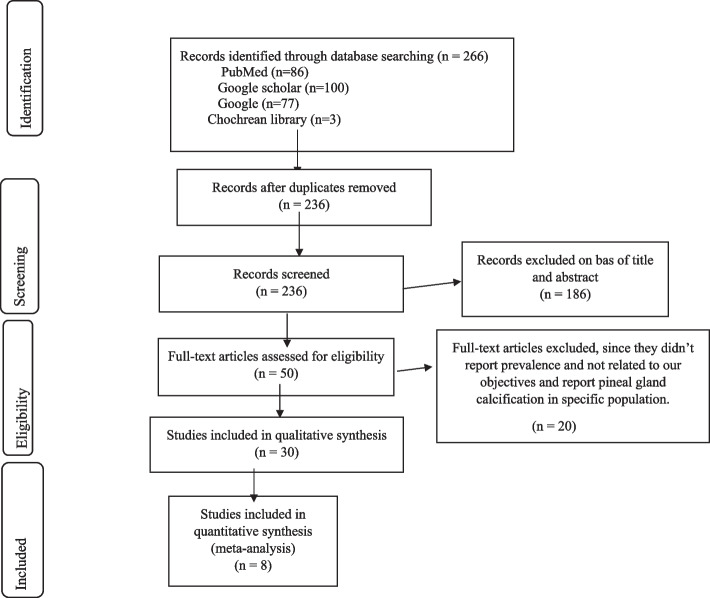
Flow diagram of studies included for the systematic review and meta-analysis

A total of 266 published articles were obtained from different electronic databases. After the removal of 30 duplicates, 236 of them were assessed based on the title and abstract. From these, only 50 publications were considered for full-text review and of these 20 studies were excluded because they either did not report the prevalence of pineal gland calcification or were done on specific population groups. Finally, 30 peer-reviewed papers were included for qualitative analysis, and after eligibility evaluation, only 8 papers were retained for the final meta-analysis, two in the USA, one in South Africa, one in India, one in Cameron, one in Iraq, one in Iran, one in Ethiopia, one in Turkey, and one in India. The study selection process and flowchart of the literature search are shown in the diagram below (Fig. 1). The prevalence of pineal gland calcification ranged from 26.88% in Iraq to 76.7% in India, and according to the qualitative analysis, an increase in age, male sex, and white ethnicity were the major socio-demographic characteristics that increase the prevalence of pineal gland calcification. The information from selected papers with their prevalence is described and presented in Table 1.

**Table 1:** The prevalence of pineal gland calcification

Author	Year	Country	Study design	Sampling technique	Sample size	Prevalence	Method of detection SYSR-D-21-00125R4
Koranteng DPN	2015	South Africa	Cross-sectional	Consquative	450	76%	CT scan
Uduma	2011	Cameron	Cross-sectional	Consquative	174	46.2%	CT scan
M.H. Daghighi et al	2007	Iran	Cross-sectional	Consquatve	1569	71%	CT scan
PP Sedghizadeh	2012	USA	Cross-sectional	Consquative	500	35.2%	CT scan
Admassie et al	2009	Ethiopia	Cross-sectional	Consquative	518	72%	CT scan
Nada Aljarba	2017	Saudi	Cross-sectional	Consquative	54	64.8%	CT scan
AhmetTuncayTurgut	2008	Turkey	Cross-sectional	Cosquative	1376	68.5%	CT scan
Sunil	2015	USA	Cross-sectional	Consquative	500	58.8%	CT scan

**Fig. 2:**
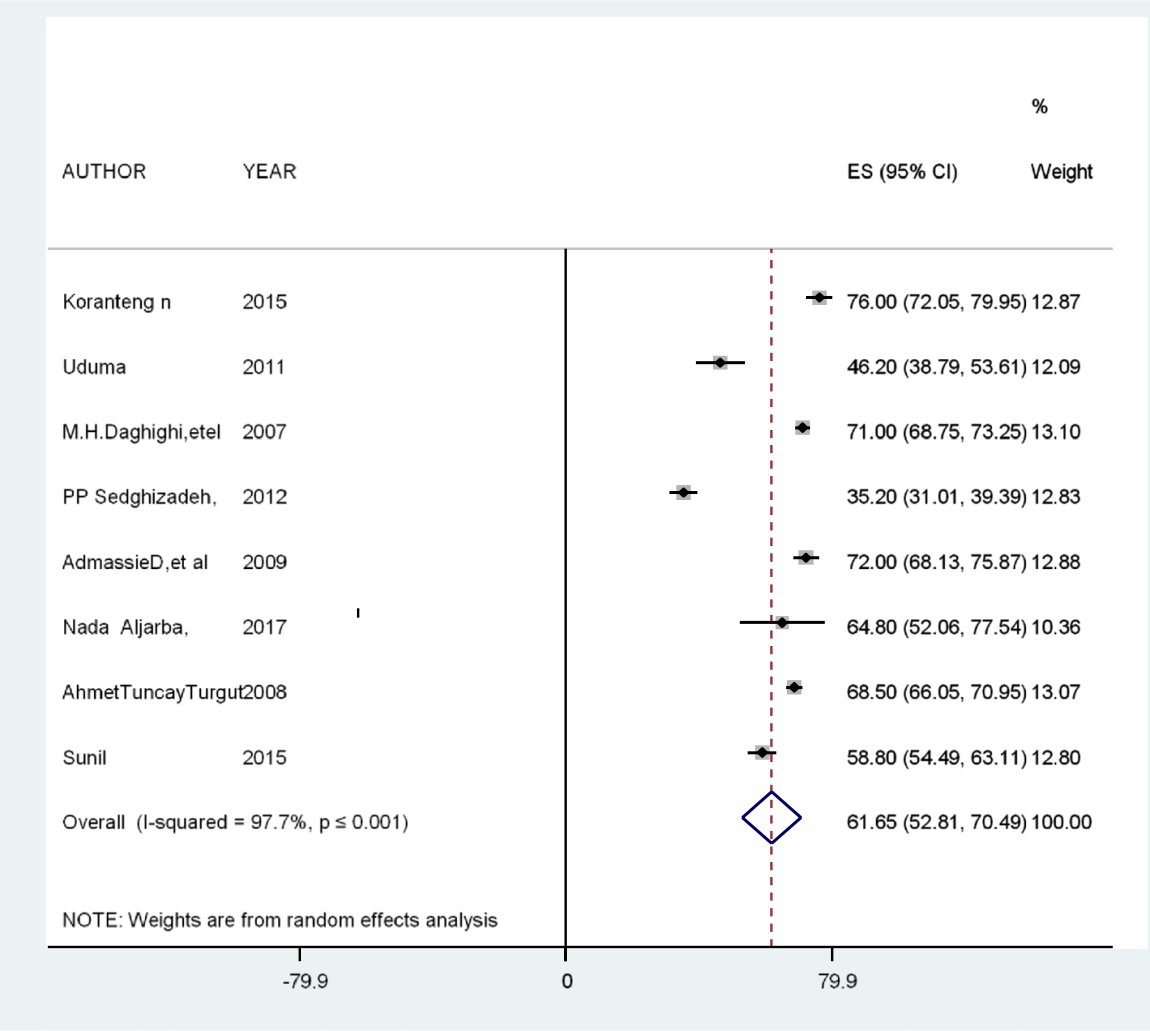
The prevalence of pineal gland calcification among different studies

Different studies showed that the prevalence of pineal gland calcification ranges from 35.2% in California to 76% in South Africa [3–5, 13, 16, 21, 24]. The pooled prevalence of pineal gland calcification in this study was 61.65% [95% CI: 52.81, 70.49], with a heterogeneity of *I*^2^ = 97.7%, *P* ≤ 0.001 (Fig. 2). A meta-regression analysis was conducted since there was statistically significant heterogeneity,* I*-square test statistics, and *P*-value less than 0.05 and to identify the source of heterogeneity and to have an appropriate interpretation of the findings. However, the meta-regression analysis found no significant variables which can explain the heterogeneity. There were no statistically significant study-level covariates: sample size, publication year, and geographical regions of the included studies. Therefore, the heterogeneity can be explained by other factors not included in this systematic review (Table 2).

**Table 2:** Meta-regression for the prevalence of pineal land calcification

Covariant	Metaregresion coefficient	*P*-value	95% CI	Variance explained
Univariable analysis				
Country	0.705	0.741	− 4.05, 5.47	− 11.25%
Year of publication	− 0.63	0.764	5.43, 4.16	12.39%
Sample size	0.007	0.588	− 0.021, 0.034	7.89%
Multivariable analysis				50.08%
Country	1.002	0.74	− 6.2973, 8.30	
Year of publication	0.38	0.92	− 9.97, 10.74	
Sample size	0.009	0.73	− 0.056, 0.075	

### Publication bias

**Fig. 3:**
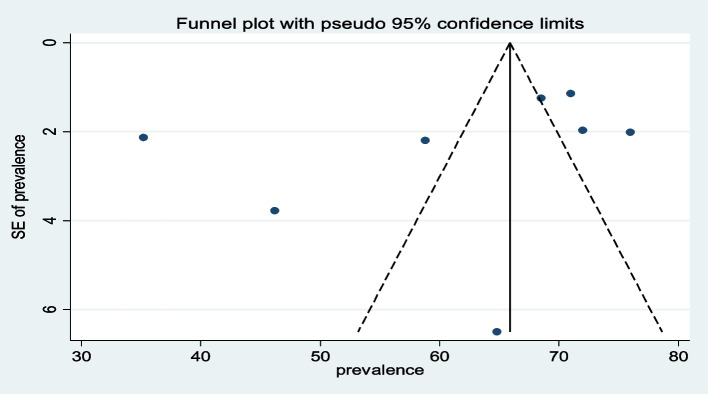
The funnel plot for the assessment of publication bias among different studies

A review of the funnel plots did not rule out the potential for publication bias of pineal gland calcification and it was assessed using Egger’s test (Fig. 3). The estimated bias coefficient was − 12.9 with a standard error of 13.04, giving a *P*-value of 0.36. Thus, the test provides no evidence for the presence of a small-study effect (*P*-value > 0.05) (Table 3).

**Table 3:** Egger’s test for the assessment of publication bias

Std_Eff	Coef	Std. Err	*T*	*P*-value	[95% Conf. interval]	
Slope	82.77653	25.15959	3.29	0.022	18.10174	147.4513
Bias	− 12.9568	13.04276	− 0.99	0.366	− 46.48427	20.57068

## Discussion

Systematic review and meta-analysis were done to estimate the pooled prevalence of pineal gland calcification using published studies. The prevalence of pineal gland calcification in this study was 61.65% [95% CI: 52.81, 70.49], with a heterogeneity of *I*^2^ = 97.7%, *P* ≤ 0.001, but different studies reported variation in the prevalence of pineal gland calcification from 35.2 to 76% [22, 25]. This regional variation of pineal gland calcification may be associated with altitude, sunlight exposure, ethnicity, nutritional variation, and neurodegenerative diseases, which are associated with the magnitude of pineal gland calcification [7]. It might also be associated with the age group of the study population included since as age increases, the metabolic activity of the gland increases, which leads to a higher degree of pineal gland calcification [2]. The prevalence of pineal gland calcification in this systematic review and meta-analysis was in line with studies conducted in Saudi Arabia, Turkey, and the USA [23, 25]; however, the prevalence in this systematic review and meta-analysis was smaller than the reports in Ethiopia and Iran [26, 27] and greater than the prevalence reported in California [22]. This regional variation of pineal gland calcification might be associated with environmental variation across different regions of the world, which is directly related to pineal gland calcification [5]. This variation of pineal gland calcification might also be associated with the sensitivity of the computed tomography (CT) scan used in the detection of pineal gland calcification. Different studies indicated that pineal gland calcification was significantly associated with age, sex, low altitude, low sunlight exposure, ethnicity, light, cell phone, fluoride intake, nutrition, and neurodegenerative diseases [7]. A modern lifestyle like using electromagnetic field (EMF)-emitting material such as cell phones and audio/video players; using fluoride in mouthwash, toothpaste, and tab water; and using herbicides were also considered as the critical risk factors for pineal gland calcification [12, 28, 29]. The prevalence of pineal gland calcification was highest in older age individuals compared with those of young and also highest among obese. Raw and organic chocolate is rich in antioxidants that fight free radicals and keep our brain healthy, promoting detoxification of the pineal gland [10]. In this study, there is a high prevalence of pineal gland calcification detected by CT scan and most studies showed that males have a high prevalence of pineal gland calicifcation [2–7]. Even though there is no clear justification for this variation of pineal gland calcification by sex of individuals, many other studies link such differences to the effect of melatonin on gonadotropins [28–30]. As a limitation, most of the studies lack clinical and laboratory data on the function of the pineal gland, making it difficult to extrapolate these findings to neurodegenerative changes in a clinically relevant way.

## Conclusion

In this study, the pooled prevalence of pineal gland calcification was higher compared with many previous reports, which is more prevalent in the adult population compared with the pediatric. According to the qualitative analysis, an increase in age, male sex, and white ethnicity are the major socio-demographic characteristics that increase the prevalence of pineal gland calcification.

## Data Availability

Not applicable.

## Abbreviations

AD: Alzheimer’s disease
ADHD: Attention-deficit/hyperactivity disorder
BBB: Blood-brain barrier
CBCT: Cone-beam computed tomography
CPV: Calcified pineal volume
CT: Computed tomography
DMLO: Dim light melatonin onset
DMLOff’s: Dim light melatonin offset
DOC: Degree of pineal gland calcification
DSPS: Delayed sleep phase syndrome
EMF: Electromagnetic field
NPV: Noncalcified pineal volume
PGC: Pineal gland calcification
POC: Proportion of calcification
TORCH: Toxoplasmosis, others, rubella, herpes zoster
TPV: Total pineal volume
USA: United States of America
